# Nonlinear response from optical bound states in the continuum

**DOI:** 10.1038/s41598-019-43672-y

**Published:** 2019-05-09

**Authors:** Evgeny N. Bulgakov, Dmitrii N. Maksimov

**Affiliations:** 1 Reshetnev Siberian State University of Science and Technology, 660037 Krasnoyarsk, Russia; 2 Kirensky Institute of Physics, Federal Research Center KSC SB RAS, 660036 Krasnoyarsk, Russia; 3 Siberian Federal University, Krasnoyarsk, 660041 Russia

**Keywords:** Photonic crystals, Nonlinear optics

## Abstract

We consider nonlinear effects in scattering of light by a periodic structure supporting optical bound states in the continuum. In the spectral vicinity of the bound states the scattered electromagnetic field is resonantly enhanced triggering optical bistability. Using coupled mode approach we derive a nonlinear equation for the amplitude of the resonant mode associated with the bound state. We show that such an equation for the isolated resonance can be easily solved yielding bistable solutions which are in quantitative agreement with the full-wave solutions of Maxwell’s equations. The coupled mode approach allowed us to cast the the problem into the form of a driven nonlinear oscillator and analyze the onset of bistability under variation of the incident wave. The results presented drastically simplify the analysis nonlinear Maxwell’s equations and, thus, can be instrumental in engineering optical response via bound states in the continuum.

## Introduction

Optical bound states in the continuum (BICs) are peculiar localized eigenstates of Maxwell’s equations embedded in the continuous spectrum of scattering solutions^1^. In the recent decade BICs have been theoretically predicted^2–10^ and experimentally observed^11–16^ in various dielectric set-ups with periodical permittivity. The BICs in photonic systems have already found important applications in enhanced optical absorbtion^17^, surface enhanced Raman spectroscopy^18^, lasing^19^, sensors^20,21^, and filtering^22^.

Spectrally, the optical BICs are points of leaky bands above the line of light where the quality factor (*Q*-factor) diverges to infinity^1^. By themselves the BICs are localized solutions decoupled from any external waves incident on the system. However, even the slightest off-set from the BICs point in the momentum space transforms the BICs into high-*Q* resonant modes with unlimited *Q*-factor as far as the material losses in the supporting structure are neglected. In other words the BICs are spectrally surrounded by strong resonances which can be excited from the far-field to arbitrary high amplitude by tuning the angle of incidence of the incoming wave^23^. The excitation of the strong resonances results in *critical field enhancement*^24,25^ with the near-field amplitude controlled by the frequency and the angle of incidence of the incoming monochromatic wave.

In this paper we investigate the role of the critical field enhancement in activation of nonlinear optical effects due to the cubic Kerr nonlinearity. The earlier studies on the nonlinear effects were mostly concentrated on the BICs supported by microcavities coupled to waveguide buried in the bulk photonic crystals, where the nonlinear effects of symmetry breaking^26^ and channel dropping^27^ were demonstrated. More recently the focus has been shift towards much simplier systems such as arrays of dielectric rods^23,28^ and dielectric gratings^29^. So far, the problem was approached from two differing directions, full-wave modelling^23,28^ that relies on exact numerical solution of Maxwell’s equations, and phenomenological coupled mode approach^29^ that employs a set of equation in form of environment coupled nonlinear oscillators. The former approach provides the solutions of the Maxwell’s equations via time expensive numerical simulations with no insight into the physical picture of the effect while the latter relies on a set of unknown parameters whose numerical values have to be specified by fitting to exact numerical solutions. Here we bring the two approaches together by deriving the coupled mode equation for the amplitude of the high-*Q* resonant mode in the spectral vicinity of the BIC. Thus, the problem is cast into the form of a single driven nonlinear oscillator. We show that all parameters such *nonlinear* coupled mode theory (CMT) can be easily derived from the solution of the *linear* scattering problem, and demonstrate the validity of our approach by comparing the CMT solutions against full-wave simulations data.

## Scattering Theory

**Figure 1:**
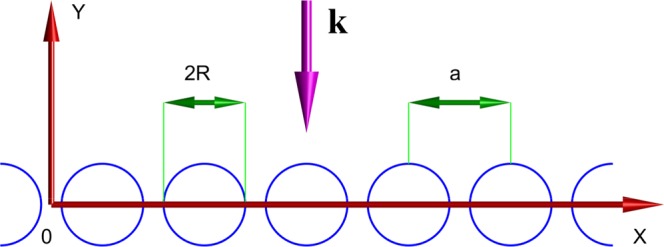
Set-up of the array in *x*0*y*-plane. The circles show the surface cross-section of dielectric rods with nonlinear permittivity. The thick magenta arrow shows the incident wave vector **k** at the normal incidence. The electric vector of the incident wave is always alighted with the *z*-axis perpendicular to the plane of the plot.

We consider an array of identical dielectric rods of radius R, arranged along the x-axis with period *a*. The axes of the rods are collinear and aligned with the *z*-axis. The cross-section of the array in x0y -plane is shown in Fig. 1. The scattering problem is controlled by Maxwell’s equation which for the further convenience are written in the matrix form as follows

1 $$\{\begin{array}{cc}0 & \nabla \times \\ -\,\nabla \times  & 0\end{array}\}\{\begin{array}{c}{\bf{E}}\\ {\bf{H}}\end{array}\}=\frac{\partial }{\partial t}\{\begin{array}{c}\varepsilon {\bf{E}}\\ {\bf{H}}\end{array}\},$$

where $\varepsilon$ is the non-linear dielectric permittivity $\varepsilon ={n}^{2}$ with *n* as the refractive index

2 $$n={n}_{0}+{n}_{2}I,$$

where *n*_0_ is the linear refractive index, *n*_2_ is the nonlinear refractive index, and $I=|{\bf{E}}{|}^{2}$ is the intensity. The scattering problem can be reduced to a single two-dimensional stationary differential equation if monochromatic incident waves propagate in the directions orthogonal to the *z*-axis. In case of *TM*-polarized waves that equation is written as

3 $$\frac{{\partial }^{2}u}{\partial {x}^{2}}+\frac{{\partial }^{2}u}{{\partial }^{2}{y}^{2}}+{k}_{0}^{2}\varepsilon u=0,$$

where *u* is the *z*-component of the electric field $u={E}_{z}$, and *k*_0_ is the vacuum wave number (frequency). Notice, that above we set the speed of light to unity to measure the frequency in the units of distance. Assuming that a plane wave is incident from the upper half-space in Fig. 1. the solution $y > R$ outside the scattering domain is written as

4 $$u(x,y)=\sqrt{2}\,\sum _{j=-\infty }^{\infty }\,{r}_{j}{e}^{i[{\alpha }_{j}x+{\beta }_{j}(y-R)]}+\sqrt{2{I}_{0}}{e}^{i[{\alpha }_{0}x-{\beta }_{0}(y-R)]},$$

where ${\alpha }_{j}={k}_{x}+2\pi j$/*a*, *I*_0_ is the intensity of the incident monochromatic wave, and ${\beta }_{j}=\sqrt{{k}_{0}^{2}-{\alpha }_{j}^{2}}$ with *k*_*x*_ as the *x*-component of the incident wave vector. In the lower half-space we have

5 $$u(x,y)=\sqrt{2}\,\sum _{j=-\infty }^{\infty }\,{t}_{j}{e}^{i[{\alpha }_{j}x-{\beta }_{j}(y+R)]}.$$

The prefactor $\sqrt{2}$ in Eqs (4 and 5) is introduced to have a unit period-averaged magnitude of the Poynting vector $\langle |{\bf{S}}|\rangle ={{\bf{E}}}^{\dagger }{\bf{E}}$/$2={I}_{0}$.

**Figure 2:**
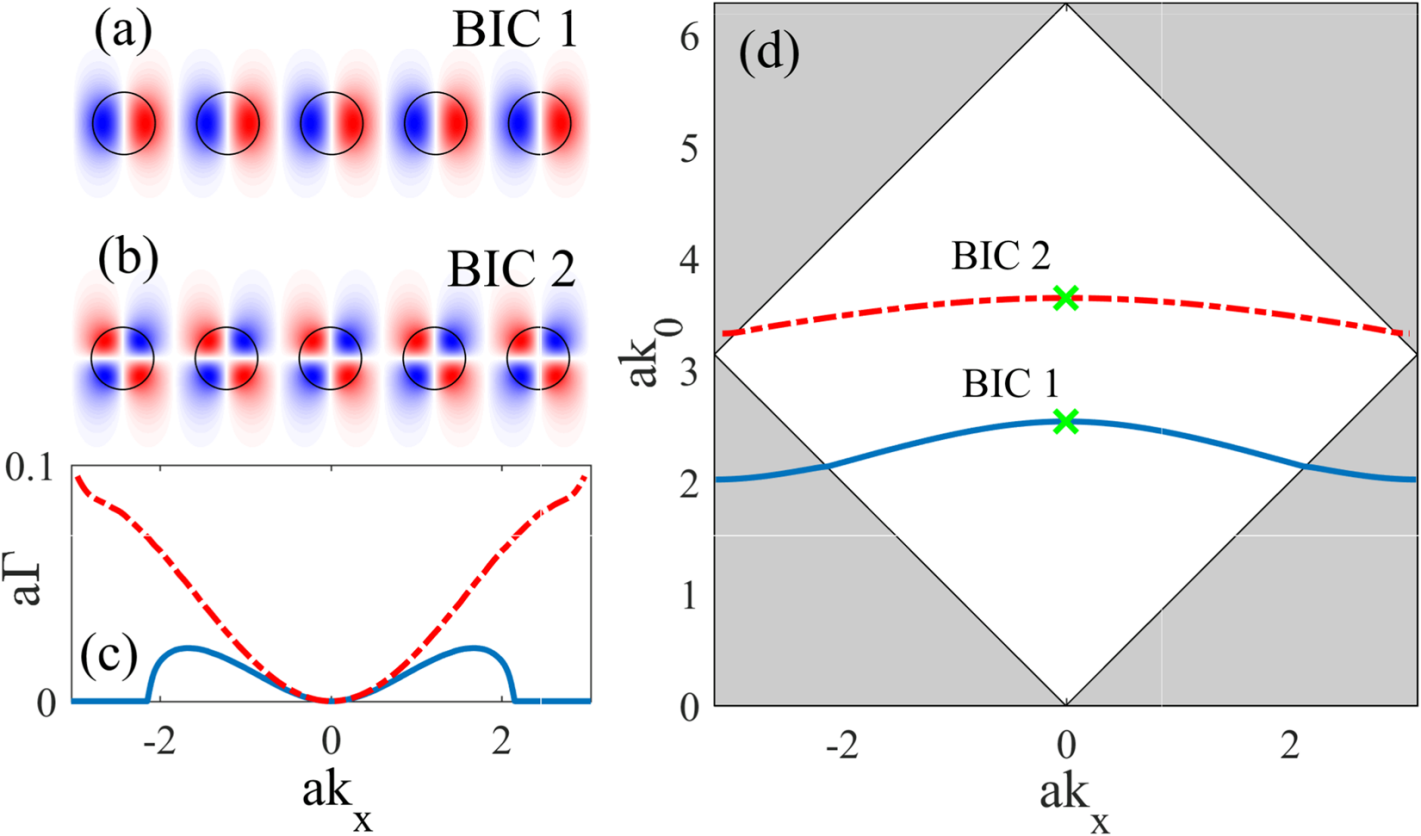
BICs in the array of dielectric rods with $R=0.3a$, and dielectric permittivity within the rods ${\varepsilon }_{1}=12$. The ambient medium is air. (**a**,**b**) The field profiles, *E*_*z*_(*x*, *y*) (p.d.u.) of BICs with eigenfrequencies $a{\bar{k}}_{BIC}=2.5421$, and $a{\bar{k}}_{BIC}=3.6468$. (**c**) The dispersion of the imaginary part of the resonant eigenvalues. (**d**) The real part of the resonant eigenvalue of the leaky zones hosting the BICs; BIC 1 - solid blue, BIC 2 - dash red. The positions of the BICs are shown by green crosses.

The solution of the scattering problem is defined by the unknowns *t*_*j*_, *r*_*j*_ in Eqs (4 and 5). Here for finding the BICs and the scattering solutions we applied a numerically efficient method based on the Dirichlet-to-Neumann maps^30,31^. We restrict ourselves with the simplest, namely, symmetry protected BICs. Such BICs occur in the ${\rm{\Gamma }}$-point as standing waves symmetrically mismatched with outgoings waves with ${k}_{x}=0$. The field profiles of two such BICs are shown in Fig. 2(a,b). The BICs are points of the leaky zones with a vanishing imaginary part of the resonant eigenvalue $\bar{k}={\bar{k}}_{0}-i\gamma$. The dispersions of the imaginary and real parts of the resonant eigenvalue are shown in Fig. 2(c,d), respectively.

**Figure 3:**
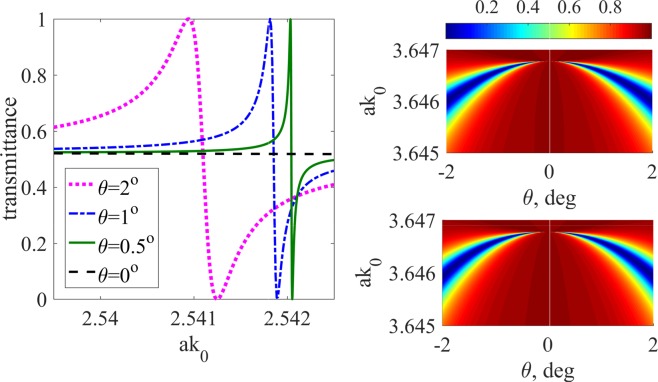
Scattering of a monochromatic plane wave in the spectral vicinity of BICs, ${n}_{0}=12$ (polycrystalline silicon at 1.8 *μ*m^48^). (Left panel) Collapsing Fano feature in transmittance in the spectral vicinity of BIC 1 from Fig. 2. (Right panel) Transmittance in the spectral vicinity of BIC 2; top - full-wave solution, bottom - CMT approximation.

One important signature of the BICs is a narrow Fano feature in the transmittance spectrum with occurs in the spectral vicinity of the BIC^32–35^ as the angle of incidence, $\theta =\arcsin ({k}_{x}/{k}_{0})$ is slightly detuned from the normal. This effect is illustrated in Fig. 3 (left panel). One can see from Fig. 3 that the BIC induces a Fano resonance that collapses on approach to the normal incidence.

To quantitatively describe the scattering in the spectral vicinity of the BICs we resort to coupled mode theory (CMT) for a single isolated resonance^36^. According to CMT the amplitude of a leaky mode *c*(*t*) obeys the following temporal equation

6 $$\frac{dc(t)}{dt}=-\,(i{\bar{k}}_{0}+\gamma )c(t)+\varkappa \sqrt{{I}_{0}}{e}^{-i{k}_{0}t},$$

where ${\bar{k}}_{0}$, *γ* are given by dispersion relationships shown in Fig. 2(c,d), and $\varkappa$ is the coupling coefficient. In the case of stationary scattering, $c(t)=c{e}^{-i{k}_{0}t}$ the scattering matrix is written as^36^

7 $$\hat{S}=\hat{C}+\frac{{\bf{d}}{{\bf{d}}}^{T}}{i({\bar{k}}_{0}-{k}_{0})+\gamma },$$

where $\hat{C}$ is the matrix of the direct process, and ${{\bf{d}}}^{T}=[\varkappa ,\pm \,\varkappa ]$, the sign +(−) being chosen if the mode is symmetric (antisymmetric) with respect $y\to -\,y$, see Fig. 2(a,b). By applying energy conservation it can be shown^36^ that ${{\bf{d}}}^{\dagger }{\bf{d}}=2\gamma$, therefore $\varkappa ={e}^{i\delta }\sqrt{\gamma }$. In addition the time reversal yields $\hat{C}{{\bf{d}}}^{\ast }=-\,{\bf{d}}$. Since $\hat{C}$ is symmetric the latter constraint uniquely defines the phase *δ*. The spectrum in Fig. 2(c,d) is symmetric with respect to ${k}_{x}\to -\,{k}_{x}$, hence in the vicinity of the symmetry protected BICs we can write^37^

8 $$\begin{array}{rcl}a{\bar{k}}_{0}(\theta ) & = & a{\bar{k}}_{BIC}+{a}_{2}{\theta }^{2}+{a}_{4}{\theta }^{4}+{\mathscr{O}}({\theta }^{6}),\\ a\gamma (\theta ) & = & {b}_{2}{\theta }^{2}+{b}_{4}{\theta }^{4}+{\mathscr{O}}({\theta }^{6}).\end{array}$$

In Table 1 we collect the values of all parameters necessary for finding transmittance and reflectance with equation (7). The parameters *a*_2_, *a*_4_, *b*_2_, *b*_4_ are extracted by the least square fit in the vicinity of the BIC, while the entries of $\hat{C}$ are found at the normal incidence and the BIC frequency of the incident wave. In Fig. 3 (right panel) we plot the transmittance in the spectral vicinity of BIC 2 obtained through full-wave modelling in comparison against the CMT fit. One can see that the CMT reproduces the full-wave solution to a good accuracy.

**Table 1:** Parameters of the scattering theory in the spectral vicinity of BIC 1 and BIC 2.

BIC	${\boldsymbol{a}}{\bar{{\boldsymbol{k}}}}_{{\boldsymbol{BIC}}}$	${\{\hat{{\boldsymbol{C}}}\}}_{{\bf{1}},{\bf{1}}}$	${\{\hat{{\boldsymbol{C}}}\}}_{{\bf{1}},{\bf{2}}}$	*a*_2_ × 10^4^	*a*_4_ × 10^6^	*b*_2_ × 10^5^	*b*_4_ × 10^8^
1	2.54211	−0.42170 − 0.55137*i*	0.57178 − 0.43730*i*	−2.5403	1.3313	3.7506	−2.7612
2	3.64678	0.15687 + 0.05864*i*	−0.34521 + 0.92346*i*	−1.6338	−8.7802	8.9939	−3.1609

## Effect of the Nonlinearity

The effect of the nonlinearity can be incorporated to the time-stationary CMT equation by introducing nonlinear frequency shift Δ*k*_0_ due to the Kerr effect

9 $$[i({\bar{k}}_{0}-{\rm{\Delta }}{k}_{0}-{k}_{0})+\gamma ]c=\varkappa \sqrt{{I}_{0}},$$

where Δ*k*_0_ is dependent on *c*. The perturbative frequency shift induced by variation of dielectric constant can be found as^38–41^

10 $${\rm{\Delta }}{k}_{0}=\frac{\lambda }{4}|c{|}^{2},$$

where

11 $$\lambda =2{n}_{0}{n}_{2}\,{\int }_{{S}_{R}}\,dS|{{\bf{E}}}_{BIC}{|}^{4},$$

with integration performed over the cross section of the dielectric rod, *S*_*R*_ and the BIC field **E**_*BIC*_ normalized to store a unit period averaged energy

12 $${\int }_{S}\,dS\frac{{n}_{0}{(x,y)}^{2}{{\bf{E}}}^{\dagger }{\bf{E}}+{{\bf{H}}}^{\dagger }{\bf{H}}}{4}=1,$$

where *S* is the area of the elementary cell. Although equation (11) is known to have certain limitations for low-*Q* cavities^42^, it is found to be applicable for high-*Q* nonlinear cavities embedded into the bulk photonic crystals^38^.

In more detail, to introduce the effect of nonlinearity into CMT we decompose the electromagnetic field into two components ${\bf{E}}={{\bf{E}}}_{res}+{{\bf{E}}}_{dir}$, ${\bf{H}}={{\bf{H}}}_{res}+{{\bf{H}}}_{dir}$. Here subscript *dir* designates the direct field contribution associated with the non-resonant optical pathway through the structure, while subscript *res* is used for the contribution due to resonant excitation of the leaky wave which evolves to a BIC at the normal incidence, see Fig. 2(c,d). Substituting the decomposed field into Maxwell’s equations, equation (1) one finds

13 $$\{\begin{array}{cc}0 & \nabla \times \\ -\,\nabla \times  & 0\end{array}\}\{\begin{array}{c}{{\bf{E}}}_{res}\\ {{\bf{H}}}_{res}\end{array}\}-\frac{\partial }{\partial t}\{\begin{array}{c}\varepsilon {{\bf{E}}}_{res}\\ {{\bf{H}}}_{res}\end{array}\}=\{\begin{array}{c}-\,\nabla \times {{\bf{H}}}_{dir}\\ \nabla \times {{\bf{E}}}_{dir}\end{array}\}+\frac{\partial }{\partial t}\{\begin{array}{c}\varepsilon {{\bf{E}}}_{dir}\\ {{\bf{H}}}_{dir}\end{array}\}.$$

The temporal dependance of the resonant contribution can be written as ${{\bf{E}}}_{res}(t)=c(t){{\bf{E}}}_{0}$, ${{\bf{H}}}_{res}(t)=c(t){{\bf{H}}}_{0}$, where **E**_0_, **H**_0_ are the electric and magnetic field profiles of the leaky mode. Multiplying from the left by 1/4$[{{\bf{E}}}_{0}^{\dagger },{{\bf{H}}}_{0}^{\dagger }]$ and integrating over the scattering domain one immediately finds

14 $$\frac{d}{dt}(c(t)+\frac{\lambda }{4}|c(t){|}^{2}c(t))=-\,(i{\bar{k}}_{0}+\gamma )c(t)+b{e}^{-i{k}_{0}t},$$

where we assumed that **E**_*dir*_, **H**_*dir*_ are monochromatic fields with frequency *k*_0_, and neglected the nonlinear effects in the direct field since its amplitude is much smaller than that of the resonant field. We also assumed that the leaky mode is normalized according to equation (12) to be consistent we our normalization of the outgoing waves Eqs (4 and 5). By comparing equation (14) against equation (6) we find

15 $$b=\varkappa \sqrt{{I}_{0}}.$$

The only problem we are left with is to correctly define *λ*. We have mentioned that equation (14) is obtained after integration over the scattering domain which is somewhat ambiguous since the boundary between the far- and near-fields can be arbitrary defined. What is worst is that the resonant eigenmodes diverge in the far-field, and therefore require a different normalization condition^43^ rather than equation (12). One may notice, however, that evaluation of *λ* in equation (14) can only involve integration over the area of the rods where the non-linearity is present. One the other hand the leaky mode is spectrally close to the BIC, hence we conjecture that the leaky mode field profile within the rods can be replaced with that of the BIC. This approach lifts the problem of the mode normalization as the BIC is a localized state square integrable over the whole space. Thus, we end up with equation (11).

After time harmonic substitution, $c(t)=c{e}^{-i{k}_{0}t}$, equation (14) can be solved for the system’s response to a monochromatic wave. The transmission amplitude can be found as^36^

16 $${t}_{0}={\{\hat{C}\}}_{1,2}\sqrt{{I}_{0}}+\varkappa c.$$

The stability of time harmonic solutions can be examined with a small perturbation approach which yields that the solution is stable if and only if the real part of both eigenvalues of the matrix

17 $$\hat{M}=\{\begin{array}{cc}1+\tfrac{\lambda |c{|}^{2}}{2} & -\tfrac{\lambda {c}^{2}}{4}\\ -\tfrac{\lambda {({c}^{\ast })}^{2}}{4} & 1+\tfrac{\lambda |c{|}^{2}}{2}\end{array}\}\{\begin{array}{cc}i{k}_{0}\tfrac{\lambda |c{|}^{2}}{2}-i({\bar{k}}_{0}-{k}_{0})-\gamma  & i{k}_{0}\tfrac{\lambda {c}^{2}}{4}\\ -i{k}_{0}\tfrac{\lambda {({c}^{\ast })}^{2}}{4} & -i{k}_{0}\tfrac{\lambda |c{|}^{2}}{2}+i({\bar{k}}_{0}-{k}_{0})-\gamma \end{array}\}$$

are non-positive.

**Figure 4:**
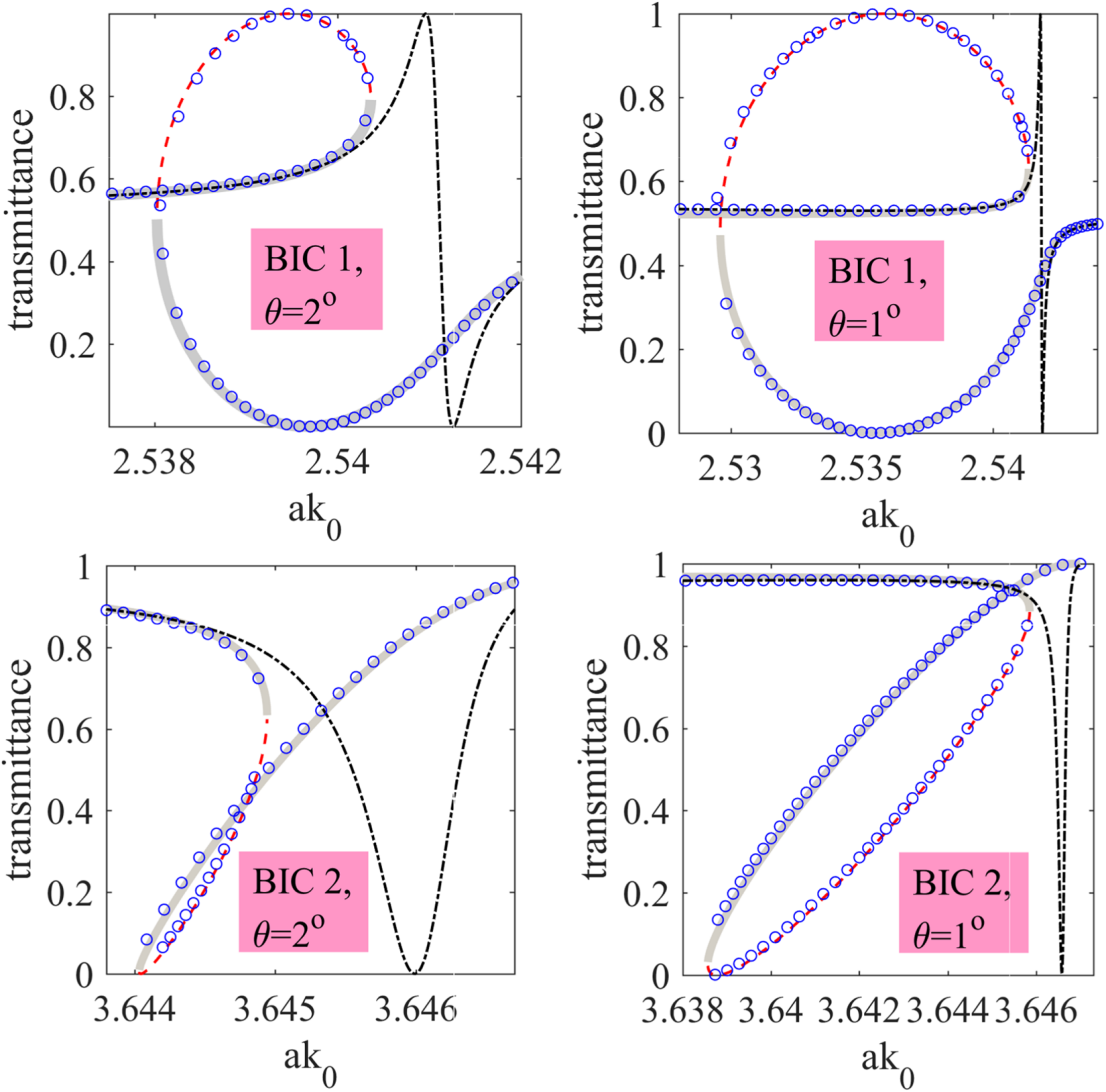
Nonlinear Fano resonance in the spectral vicinity of BIC 1 and BIC 2 at different angles of incidence, *θ* for *I*_0_ = 8.(3) MW/cm^2^. Blue circles - numerical results by Fourier-Chebyshev pseudospectral method, thick gray line - stable CMT solution, thin dash red line - unstable CMT solution, dash-dot black line - Fano line-shape unperturbed by the nonlinearity.

**Figure 5:**
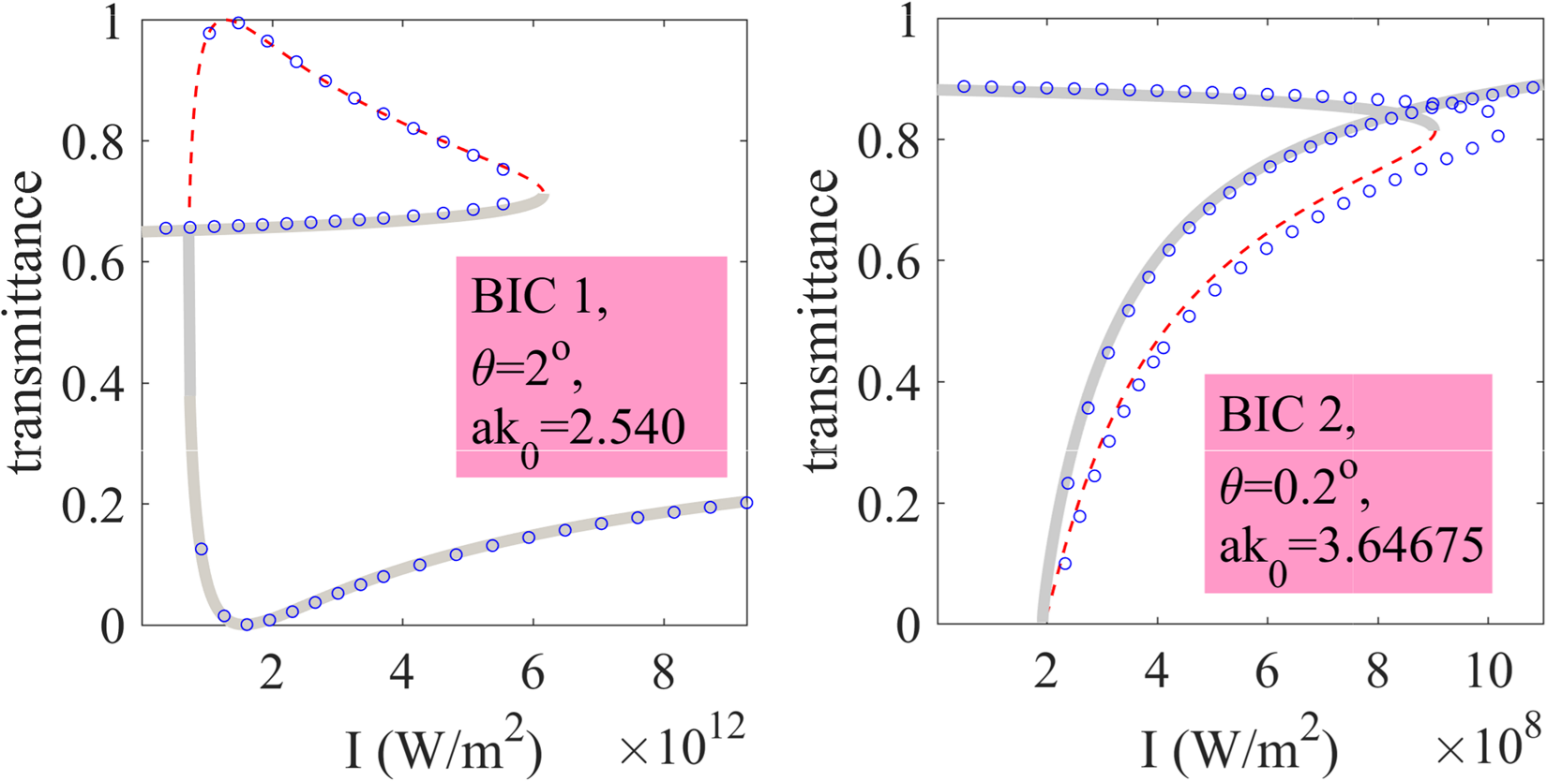
Optical bistability in the intensity domain with BIC 1 and BIC 2. Blue circles - numerical results by Fourier-Chebyshev pseudospectral method, thick gray line - stable CMT solution, thin dash red line - unstable CMT solution.

Finally, we verified our findings by comparing the solution of equation (14) against exact numerical solutions of equation (3) obtained with Fourier-Chebyshev pseudospectral method^44^. For our numerical simulations we took ${n}_{2}=5\times {10}^{-18}\,{{\rm{m}}}^{2}$/W which corresponds to silicon at 1.8 *μ*m^45^. The results are shown in Fig. 4 where one can see a good agreement between the two approaches. In Fig. 4 one can see the typical picture of nonlinear Fano resonances^46^ with optical bistability triggered by critical field enhancement in the spectral vicinity of a BIC^24,25^. Notice that the stability pattern is identical to that previously reported in the literature^29,46^. We also investigated the emergence of optical bistability in the intensity domain. The simulations were again performed by both solving equation (14), and solving equation (3) by full-wave Fourier-Chebyshev pseudospectral method. In Fig. 5 (left panel) we show a picture of optical bistability in the spectral vicinity of BIC 1. Notice, that the bistability widow occurs at the intensities unobtainable with 1 *W* continuous lasers. To reduce the bistability threshold one can tune the angle of incidence approaching the BIC in the momentum space and, thus, increasing the *Q*-factor of the leaky mode^23^. This idea is exemplified in Fig. 5 (right panel) where we plot the transmittance in the spectral vicinity of BIC 2 at the incident angle $\theta =0.2\,{\rm{\deg }}$. One can see that the window of optical bistability is now (0.3 − 1.5) × 10^4^ W/cm^2^.

The bistability threshold can be accessed by equating the resonant width *γ* to the frequency shift induced by the nonlinearity Δ*k*_0_ at the spectral point of maximal resonant enhancement. That yields $a\gamma =(\lambda /4){I}_{0}/(a\gamma )$. By applying equation (8) up to the term quadratic in *θ* one finds

18 $${I}_{0}=\frac{4}{\lambda }{b}_{2}^{2}{\theta }^{4}.$$

One can see from equation (18) that as far as the material losses are neglected there is no intensity threshold for optical bistability induced by BICs. This result is, however, achieved at the cost of a precise control of the frequency of the incident wave so that the line width of the continuous laser has to smaller than the resonant width *γ*, hence we have seven significant digits in the inset in Fig. 5 (right panel). Theoretically, any arbitrary low threshold of optical bistability can be achieved by decreasing the angle of incidence once material losses, thermooptical effects and structure fabrication inaccuracies are neglected. In a realistic physical experiment, though, engineering optical set-ups for observing bistability with a BIC will always be a trade-off between the line width and the intensity of the laser available, as well as, should take into account thermal deformation of the structure due to heating and fabrication inaccuracies limitations on the *Q*-factor.

## Discussion

We have theoretically shown the effect optical bistablity with bound states in the continuum (BIC). The physical picture of the effect is explained through coupled mode theory which allowed us to cast the problem of optical response to the simple form of a single driven nonlinear oscillator. That problem, although with some efforts, can be approached analytically^46,47^. The proposed coupled mode approach reduces the problem of nonlinear response to finding the solution of the *linear* Maxwell’s equation in the spectral vicinity of the BIC. Then, all parameters entering the *nonlinear* coupled mode equation can be easily found from the dispersion of the leaky band hosting the BIC, the scattering matrix of the direct process, and the BIC mode profile. The proposed method enormously simplifies analyzing the nonlinear effects induced by bound states in the continuum since it makes possible to avoid time expensive full-wave simulations. The resulting picture of a nonlinear Fano resonance can be easily understood in terms of a frequency shift due to the Kerr nonlinearity activated by critical field enhancement in the spectral vicinity of a BIC. We believe that the results will be of use in engineering optical set-ups for observation nonlinear effects with BICs.

## Data Availability

The data that support the findings of this study are available from the corresponding author, D.N.M., upon reasonable request.
